# Efficacy and Safety of Danirixin (GSK1325756) Co-administered With Standard-of-Care Antiviral (Oseltamivir): A Phase 2b, Global, Randomized Study of Adults Hospitalized With Influenza

**DOI:** 10.1093/ofid/ofz163

**Published:** 2019-04-03

**Authors:** Anuradha Madan, Shuguang Chen, Phillip Yates, Michael L Washburn, Grace Roberts, Andrew J Peat, Yu Tao, Michael F Parry, Otis Barnum, Micah T McClain, Sumita Roy-Ghanta

**Affiliations:** 1 GlaxoSmithKline, Upper Providence, Pennsylvania; 2 GlaxoSmithKline, Stevenage, United Kingdom; 3 GlaxoSmithKline, Research Triangle Park, North Carolina; 4 Stamford Hospital, Stamford, Connecticut; 5 Natchitoches Regional Medical Center, Natchitoches, Louisiana; 6 Duke University Center for Applied Genomics and Precision Medicine, Durham, North Carolina

**Keywords:** CXCR2 antagonist, danirixin, hospitalization, influenza, neutrophils

## Abstract

**Background:**

Excessive neutrophil migration has been correlated with influenza symptom severity. Danirixin (GSK1325756), a selective and reversible antagonist of C-X-C chemokine receptor 2, decreases neutrophil activation and transmigration to areas of inflammation. This study evaluated the efficacy and safety of intravenous (IV) danirixin co-administered with oseltamivir for the treatment of adults hospitalized with influenza.

**Methods:**

In this phase 2b, double-blind, 3-arm study (NCT02927431), influenza-positive participants were randomized 2:2:1 to receive danirixin 15mg intravenously (IV) twice daily (bid) + oral oseltamivir 75mg bid (OSV), danirixin 50mg IV bid + OSV, or placebo IV bid + OSV, for up to 5 days. The primary endpoint was time to clinical response (TTCR).

**Results:**

In total, 10 participants received study treatment (danirixin 15mg + OSV, n = 4; danirixin 50mg + OSV, n = 4; placebo + OSV, n = 2) before the study was terminated early due to low enrollment. All participants achieved a clinical response. Median (95% confidence interval) TTCR was 4.53 days (2.95, 5.71) for danirixin 15mg + OSV, 4.76 days (2.71, 5.25) for danirixin 50mg + OSV, and 1.33 days (0.71, 1.95) for placebo + OSV. Adverse events (AEs) were generally of mild or moderate intensity; no serious AEs were considered treatment-related. Interleukin-8 levels increased in nasal samples (using synthetic absorptive matrix strips) and decreased serum neutrophil-elastase–mediated degradation of elastin decreased in danirixin-treated participants, suggesting effective target engagement.

**Conclusions:**

Interpretation of efficacy results is restricted by the low participant numbers. The safety and tolerability profile of danirixin was consistent with previous studies.

**Clinical trial information:**

The registration data for the trial are in the ClinicalTrials.gov database, number NCT02927431, and in the EU Clinical Trials Register (https://www.clinicaltrialsregister.eu/) as GSK study 201023, EudraCT 2016-002512-40. Anonymized individual participant data and study documents can be requested for further research from www.clinicalstudydatarequest.com.

## Teaser

In this phase 2b, randomized, placebo-controlled study of intravenous danirixin in hospitalized influenza patients, all participants achieved a clinical response; danirixin had an acceptable safety profile. Early termination due to low recruitment and small sample size limits interpretation.

During the 2016–17 influenza season in the United States, a total of 18 184 laboratory-confirmed influenza-related hospitalizations were reported, with a cumulative incidence for all age groups of 65 per 100 000 population [1]. The investigation of novel treatments to reduce severity of disease and length of time spent in hospital, which in turn can reduce healthcare burden, is thus merited. One promising therapeutic approach is to target overactive and harmful aspects of the host response to influenza viruses. Neutrophils are the most abundant cells that migrate to the lungs following influenza virus infection, and excessive migration has been demonstrated to cause lung damage through the release of tissue-destructive enzymes and reactive oxygen species and the formation of neutrophil extracellular traps [2]. Levels of neutrophils or chemokines, or both, involved in neutrophil recruitment in the nasal or bronchoalveolar lavage fluid are correlated with clinical symptom severity of influenza infection in humans [3, 4].

Danirixin (GSK1325756) is a selective and reversible antagonist of the C-X-C chemokine receptor 2 (CXCR2), which is expressed on the surface of neutrophils [5]. In preclinical studies, CXCR2 antagonism has been shown to decrease neutrophil activation and transmigration to areas of inflammation [6]. Phase 1 studies in healthy adults showed that danirixin generally was tolerated well at single doses up to 400 mg and with once-daily repeat dosing for 14 days at doses of 50 mg and 200 mg, all administered orally [7]. Oral danirixin also has been evaluated in a phase 2 outpatient study in adults with acute uncomplicated influenza, given either as monotherapy or in combination with the neuraminidase inhibitor, oseltamivir, a current standard-of-care antiviral therapy [8]. In addition to the phase 2 outpatient study, further phase 2 studies have been conducted (and are ongoing) in patients with chronic obstructive pulmonary disease (COPD), which have provided a sufficient body of evidence on the safety profile of danirixin, providing support for its evaluation in a critically ill population hospitalized with influenza [8, 9].

The objective of the current study was to investigate the efficacy and safety of intravenous (IV) danirixin when co-administered with oral oseltamivir for the treatment of adults hospitalized with influenza. This is the first study in which hospitalized influenza patients were treated with IV danirixin.

## METHODS

### Study Design

**Figure 1.**
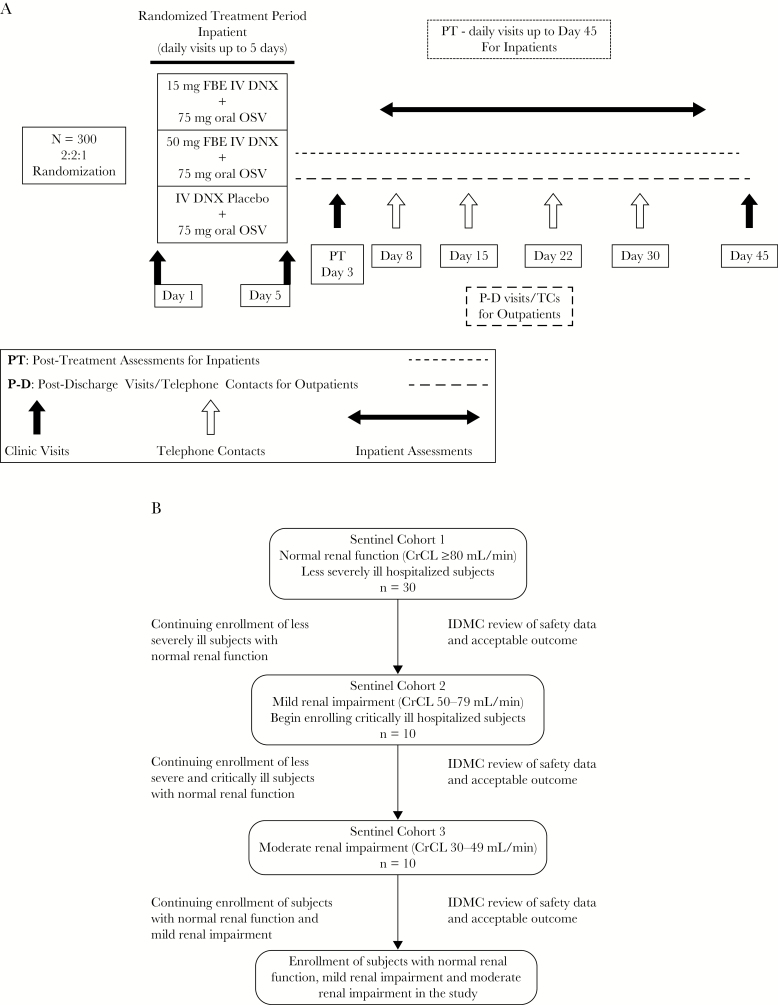
Study Design and Enrollment Cohorts. **A**, Study design. **B**, Participant enrollment and evaluation. Less severely ill hospitalized participants were defined as those with a hemodynamically stable status, requiring oxygenation with face mask, face tent, or nasal cannula, with or without radiological signs of lower respiratory tract disease or exacerbation of underlying chronic disease, including asthma, chronic obstructive pulmonary disease, or other cardiovascular conditions not leading to hemodynamic compromise. Critically ill hospitalized participants were defined as those requiring continuous positive airway pressure, bi-level positive airway pressure or mechanical ventilation, with hemodynamic instability (with or without pressor support) or illness with CNS involvement (eg, encephalopathy or encephalitis). CrCL indicates creatinine clearance; IDMC, Independent Data Monitoring Committee.

The Danirixin in Hospitalized Influenza (DAHLIA) study was a phase 2b, randomized, double-blind, placebo-controlled, 3-arm study of adult participants hospitalized with influenza (GlaxoSmithKline study 201023, EudraCT 2016-002512-40, and NCT02927431). Participants were randomized in a 2:2:1 ratio to receive danirixin 15 mg IV twice daily (bid) + oral oseltamivir 75 mg bid (hereafter referred to as the danirixin 15 mg + OSV group), danirixin 50 mg IV bid + oral oseltamivir 75 mg bid (the danirixin 50 mg + OSV group), or placebo IV bid + oral oseltamivir 75 mg bid (the placebo + OSV group). Treatment duration was up to 5 days, after which the investigator could elect to continue treatment with oral OSV. Follow-up continued until Day 45 for all participants (Figure 1A). Participants were enrolled in a stepwise manner, using 3 sentinel cohorts with increasing levels of renal impairment and disease severity, for enhanced safety monitoring (Figure 1B). The results from each cohort were to be reviewed by an independent data monitoring committee at regular intervals and before enrollment of subsequent sentinel cohorts. Participants were to be enrolled over 3 influenza seasons. However, the study was terminated after 1 season due to low enrollment.

Written informed consent was obtained from each participant. The study was conducted in accordance with the International Conference on Harmonisation of Technical Requirements for Pharmaceuticals for Human Use Guideline for Good Clinical Practice, applicable country-specific requirements, and the Declaration of Helsinki. The study was approved by the appropriate institutional review boards and independent ethics committees.

### Randomization and Blinding

Participants and site staff were blinded to danirixin and the placebo, but OSV was given open-label in all treatment arms. Participants were assigned to study treatment in accordance with the randomization schedule generated by GlaxoSmithKline (GSK) prior to the start of the study using validated internal software.

### Participant Population

Eligible participants were required to have onset of influenza symptoms within 6 days prior to study enrollment, to have required hospitalization for treatment and supportive care for influenza, and to have tested positive for influenza by a next-generation rapid reverse transcriptase-polymerase chain reaction (RT-PCR) test or other molecular-based assay.

Additional eligibility criteria were as follows: age ≥18 years; presence of fever at baseline, indicated by ≥38.0°C/≥100.4°F, or history of fever during the prior 48 hours; oxygen saturation <95% on room air by transcutaneous method, or the need for supplemental oxygen or ventilator support, or increase in oxygen supplementation requirement of ≥2 liters for participants with chronic oxygen dependency, or an oxygen saturation of at least 3% below the participant’s historical baseline oxygen saturation for those with a history of chronic hypoxia (without supplemental oxygen). At least 2 of the following were also required: respiratory rate >24 breaths/min, heart rate >100 beats/min, or systolic blood pressure (BP) <90 mm Hg.

Baseline renal criteria were as shown in Figure 1B. Baseline liver function test eligibility criteria were bilirubin ≤2× upper limit of normal (ULN) with alanine aminotransferase (ALT) ≤5× ULN or ALT >5–≤8× ULN if bilirubin <1.5× ULN.

### Endpoints and Outcome Measures

The primary endpoint was time to clinical response (TTCR), defined as hospital discharge due to clinical improvement or normalization of temperature and oxygen saturation (≥95%, without supplemental oxygen), with 2 out of 3 of the following parameters also normalized: respiratory status (return to pre-morbid oxygen requirement in participants with chronic oxygen use, reversal of need for supplemental oxygen, or respiratory rate ≤24 breaths/min without supplemental oxygen); heart rate ≤100 beats/min; or systolic BP ≥90 mmHg. Normalization of all these parameters had to be maintained for 24 hours or confirmed by hospital discharge.

Key secondary endpoints included time to respiratory response (TTRR), defined as a return to pre-morbid oxygen requirement (in participants with chronic oxygen use), a return to no requirement of supplemental oxygen, or a respiratory rate ≤24 breaths/min (without supplemental oxygen); clinical measures of influenza illness, including antibiotic use; safety and tolerability, including frequency of adverse events (AEs), serious AEs (SAEs), and change from baseline in clinical laboratory and electrocardiogram parameters.

Key exploratory endpoints included change in influenza viral load as determined by quantitative RT-PCR, assessment of co-infection as determined by multiplex RT-PCR (BioFire Diagnostics, Salt Lake City, UT), and evaluation of biomarkers of inflammation and immune response, including but not limited to interleukin (IL)-8, inducible protein (IP)-10, myeloperoxidase, neutrophil elastase and matrix metalloprotease (MMP) degraded type I collagen (C1M), MMP-degraded type III collagen (C3M), specific fragment human neutrophil-elastase–mediated degradation of elastin (EL-NE), surfactant protein D (SP-D), and soluble receptor for advanced glycoprotein end product (sRAGE) from nasal samples or fluid, whole blood, bronchoalveolar lavage, or serum, or some combination therein.

The following samples were collected throughout the study: nasal swab samples, nasopharyngeal swab samples, nasal swab samples using synthetic absorptive matrix (SAM) strips (Hunt Developments Ltd, West Sussex, UK), and optional nasal washes, whole blood samples, serum, and bronchoalveolar lavage (BAL) samples from participants where part of routine management. Nasopharyngeal samples were used for diagnosis, subtyping, and quantification. Nasal swab samples were used for diagnosis. BAL, nasal SAM strips, and nasal wash samples were used for biomarker analysis.

### Statistical Methods

Approximately 300 participants were planned to be enrolled in the study over 3 influenza seasons, with approximately 100 participants per season. This sample size was selected to achieve ≥80% overall power to detect a difference, under an assumption of a hazard ratio of 1.2 for danirixin 15 mg and 1.5 for danirixin 50 mg, and to retain a type I error <10% under the no-effect assumption.

Analyses of participant disposition, baseline characteristics, and safety analyses used the intent-to-treat exposed (ITT-E) and safety populations, which consisted of all randomized participants who received ≥1 dose of investigational product. The influenza-positive (IP) population consisted of all participants in the ITT-E population with confirmed influenza infection and was used as the primary population for the primary endpoint and all other efficacy analyses.

This study was terminated early due to only a small number of participants having been treated; thus, no formal hypothesis testing was performed. All analyses are descriptive and are not intended for making definitive conclusions. Median TTCR in each treatment arm was determined from Kaplan–Meier (KM) analyses. Due to limited data, median TTRR could not be calculated for any of the treatment arms.

Information on GSK’s data sharing commitments and requesting access can be found at https://www.clinical studydatarequest.com/.

## RESULTS

### Study Population and Participant Disposition

Although the study was planned to take place across multiple sites in multiple countries (including France, the Netherlands, the Republic of Korea, Romania, the Russian Federation, Spain, Sweden, and the United States), participants were enrolled from 5 sites in the United States and 1 each in Romania and Sweden. Enrollment occurred between January 19 and May 24, 2017 (the date of the last participant visit). Eleven participants were randomized into the study, of whom 10 received ≥1 dose of study medication; 1 participant was randomized but did not receive treatment owing to testing negative for influenza. All 10 participants were included in the ITT-E, IP, and safety populations. Participant numbers for the 3 groups were as follows: danirixin 15 mg + OSV, n = 4; danirixin 50 mg + OSV, n = 4; and placebo + OSV, n = 2.

Six participants completed the 5-day study treatment. Four participants received fewer than 5 days’ treatment because of clinical improvement, as determined by the investigator: 1 participant in the placebo + OSV arm was discharged from hospital on Day 2, and 3 participants in the danirixin + OSV arms were discharged from hospital on Day 4 or Day 5 (prior to the last danirixin bid dose). There was 1 withdrawal of the 10 ITT-E participants from the danirixin 50 mg + OSV treatment arm during follow-up, because the participant withdrew consent on Day 15. None of the placebo participants had received OSV or steroids prior to study entry for influenza symptoms. One participant in the 15 mg OSV group and 2 in the 50 mg + OSV group received OSV as prior anti-influenza therapy, and 1 participant each in the danirixin groups received steroids for their symptoms prior to study entry. Participants in the placebo group had fewer influenza symptoms at entry into the study whereas participant in the danirixin + OSV groups reported increased symptoms at study entry (Supplementary Table 1).

Baseline demographic characteristics for the 10 participants were as follows: median (min, max) age was 68.5 (34, 90) years, 60% of participants were female, and the majority (70%) of participants were of white/Caucasian/European heritage.

### Time to Clinical Response

All 10 (100%) participants in the IP population achieved a protocol-defined clinical response, with clinical improvement leading to discharge from hospital or vital sign resolution that was maintained for 24 hours. Median (95% confidence interval) TTCR by KM estimation was 4.53 (2.95, 5.71) days for danirixin 15 mg + OSV, 4.76 (2.71, 5.25) days for danirixin 50 mg + OSV, and 1.33 (0.71, 1.95) days for placebo + OSV (Table 1). Results for individual participants showed that 7 participants in the danirixin + OSV groups achieved TTCR between >2 and <6 days, and 2 participants in the placebo + OSV group achieved TTCR in ≤2 days. One participant in the danirixin 15 mg + OSV group achieved TTCR (vital sign resolution) at baseline after determination of eligibility and receiving the first dose (counted as achieving clinical response but not included in the KM estimate for TTCR).

**Table 1.** Clinical Response in the Influenza-Positive Population

	Placebo + oseltamivir 75 mg (n = 2)	Danirixin 15 mg + oseltamivir 75 mg (n = 4)^a^	Danirixin 50 mg + oseltamivir 75 mg (n = 4)^b^
**Clinical Response, n**	2	4	4
Yes, n (%)	2 (100)	4 (100)	4 (100)
Hospital discharge due to clinical improvement, n (%)	0	1 (25)	2 (50)
Hospital discharge as 24-hr confirmation,^c^ n (%)	1 (50)	2 (50)	1 (25)
Vital signs resolved, n (%)	1 (50)	1 (25)	1 (25)
**Clinical Outcome, n**	2	4	4
Clinical improvement, n (%)	2 (100)	4 (100)	4 (100)
**Kaplan–Meier Estimate, n**	2	3	4
Median, days	1.33	4.53	4.76
95% confidence interval	0.71, 1.95	2.95, 5.71	2.71, 5.25
25%–75%	0.71–1.95	2.95–5.71	3.66–5.08

^a^One participant in the danirixin 15 mg + oseltamivir 75 mg group had vital sign resolution at baseline and was counted as having a clinical response but was not included in the Kaplan–Meier estimates.

^b^One participant in the danirixin 50 mg + oseltamivir 75 mg group received oseltamivir 30 mg.

^c^Hospital discharge due to clinical improvement served as 24-hour confirmation for the clinical response.

### Time to Respiratory Response

Five (50%) participants in the IP population achieved a protocol-defined respiratory response, with 50% of participants in all groups returning to no requirement for supplemental oxygen (Table 2).

**Table 2.** Respiratory Response in the Influenza-Positive Population

	Placebo + oseltamivir 75 mg (n = 2)	Danirixin 15 mg + oseltamivir 75 mg (n = 4)	Danirixin 50 mg + oseltamivir 75 mg (n = 4)^a^
**Positive Respiratory Response, n**	2	4	4
Yes, n (%)	1 (50)	2 (50)	2 (50)
Respiratory rate ≤24/min (without supplemental oxygen)	0	0	0
Return to no requirement of supplemental oxygen, n (%)	1 (50)	2 (50)	2 (50)
Return to pre-morbid oxygen (participants with chronic oxygen use)	0	0	0

^a^One participant in the danirixin 50 mg + oseltamivir 75 mg group received oseltamivir 30 mg.

### Clinical Measures of Influenza-Related Complications

One participant from each treatment arm received concomitant antibiotics (benzylpenicillin sodium, ceftriaxone, or levofloxacin) for the treatment of influenza complications. Of these 3 participants, 1 received antibiotics starting 1 day before study Day 1, 1 participant starting on Day 1, and 1 participant starting on Day 2. One participant in the danirixin 50 mg + OSV group entered the study on mechanical ventilation due to a prior chronic condition and received bi-level positive airway pressure (BIPAP) on Day 20. A second participant, in the placebo + OSV group, received BIPAP on Day 2.

### Safety

No AEs were reported for the 2 participants in the placebo + OSV arm. All participants in the danirixin + OSV treatment arms experienced 1 or more AEs (Table 3), all of which were of mild/Grade 1 or moderate/Grade 2 intensity. Laboratory assessments considered clinically significant by investigators were to be reported as AEs. No AEs of neutropenia were reported. One participant in the 50 mg danirixin +OSV group experienced neutropenia based on central laboratory results 3 days after end of treatment. The same participant reported a fungal infection, starting 2 days after the last dose of treatment, resolving in 4 days. This participant, who had a history of asthma, also reported bronchitis, which started 41 days after the last dose of treatment, and from which they were recovering at the end of the study (Day 45). This participant had 1 on-therapy AE of sinusitis on the second day of treatment, which resolved by Day 7. One participant in the danirixin 50 mg + OSV group experienced an AE of monocytosis, which started 6 days after the start of study medication and was classed by the investigator to be related possibly to study medication. This participant, who entered the study with a history of tracheostomy for mechanical ventilation, *Clostridium difficile* infection, and oxygen supplementation also had recurrence of *C. difficile* infection post-treatment on Day 8 and Day 33. One participant receiving danirixin 15 mg + OSV reported an AE of bacterial pneumonia, 1 day after the last dose, which was considered neither serious nor related to treatment by study investigators.

**Table 3.** Adverse Events Reported by Overall Frequency in the Safety Population

Preferred Term	Placebo + oseltamivir 75 mg (n = 2)	Danirixin 15 mg + oseltamivir 75 mg (n = 4)	Danirixin 50 mg + oseltamivir 75 mg^a^ (n = 4)	Total (N = 10)
Any event, n (%)	0	4 (100)	4 (100)	8 (80)
Asthma	0	1 (25)	0	1 (10)
Atelectasis	0	0	1 (25)	1 (10)
Bronchitis	0	0	1 (25)	1 (10)
Cardiac failure	0	0	1 (25)	1 (10)
Chills	0	0	1 (25)	1 (10)
COPD	0	0	1 (25)	1 (10)
*Clostridium difficile* infection	0	0	1 (25)	1 (10)
Contusion	0	1 (25)	0	1 (10)
Cough	0	1 (25)	0	1 (10)
Dyspnea	0	0	1 (25)	1 (10)
Fungal infection	0	0	1 (25)	1 (10)
Hemoglobin decreased	0	0	1 (25)	1 (10)
Hyperglycemia	0	1 (25)	0	1 (10)
Hypokalemia	0	0	1 (25)	1 (10)
Infusion-site extravasation	0	0	1 (25)	1 (10)
Monocytosis	0	0	1 (25)	1 (10)
Pneumonia bacterial	0	1 (25)	0	1 (10)
Sinusitis	0	0	1 (25)	1 (10)
Vertigo	0	1 (25)	0	1 (10)

Abbreviation: COPD, chronic obstructive pulmonary disease.

^a^One participant in the danirixin 50 mg + oseltamivir 75 mg group received oseltamivir 30 mg.

Two participants in the danirixin 50 mg + OSV treatment arm reported non-fatal SAEs considered by the investigators not to be related to study medication: aggravated heart failure in 1 participant with a history of heart failure and atrial fibrillation in 1 participant and COPD exacerbation in the other participant. Both participants required hospital readmission on Day 8 for these conditions; both recovered and were discharged on Days 13 and 11, respectively. There were no fatal AEs or any AEs that led to withdrawal from the study or discontinuation of treatment.

### Exploratory Endpoints

#### Virology

 Similar reductions in viral load occurred across all treatment arms (Supplementary Figure 1). All participants were infected with either influenza A or B virus, and no participants were found to be co-infected with other viruses. Two neuraminidase resistance-associated substitutions (S245N and V149A) were detected in 7 and 1 participants, respectively, at baseline (prior to receipt of study medication). No participants harbored the most common neuraminidase OSV resistance substitution, H275Y.

#### Biomarkers

**Figure 2.**
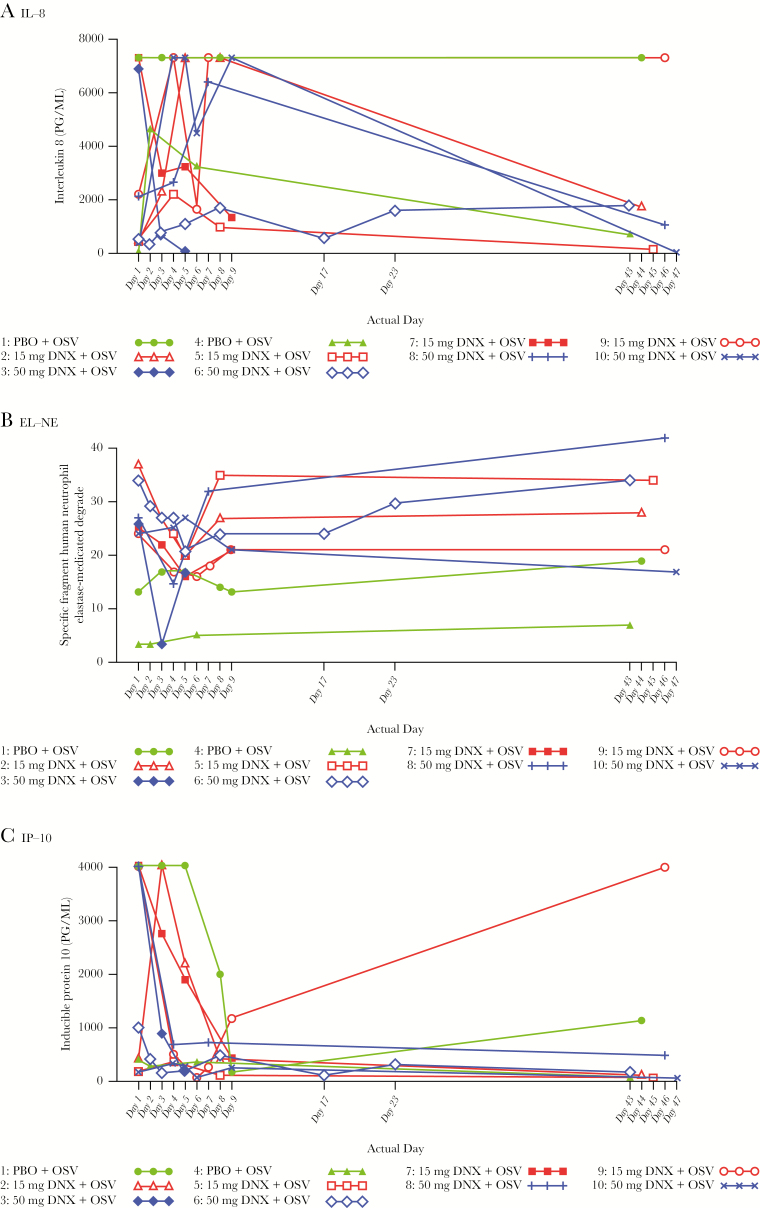
Individual Plots of Nasal SAM Strips Biomarkers by Visit and Participant: **(A)** IL-8**, (B)** EL-NE and **(C)** IP-10. A, IL-8; B, EL-NE; C, IP-10. EL-NE indicates specific fragment human neutrophil elastase mediated degradation of elastin; IL-8, interleukin (IL)-8; IP-10, inducible protein-10.

 IL-8 levels increased in nasal SAM samples during treatment through Day 5 in danirixin + OSV-treated participants and declined towards physiological baseline levels by Day 43–47. One placebo-treated participant also had an increase in IL-8 and the other placebo-treated participant had a high baseline level of IL-8 that was maintained over time (Figure 2A). No trends were observed in IL-8 nasal wash samples due to limited samples being collected.

Lung-damage–associated biomarkers C1M, C3M, EL-NE, SP-D, and sRAGE were detectable in all matrixes analyzed. A trend of decreasing serum EL-NE in both danirixin + OSV-treated groups was observed (Figure 2B), indicating fewer neutrophil- elastase–driven lung elastin degradation events, but interpretation is limited due to the small participant population. No trends in the small set of biomarkers tested were observed between treatment arms for other lung damage associated biomarkers. High levels of IP-10 were observed at baseline, which declined over time (Figure 2C).

## DISCUSSION

Of the 10 evaluable participants in this small phase 2b study, all 10 achieved a clinical response leading to hospital discharge or vital sign resolution that was maintained for 24 hours. Five of the 10 participants achieved a respiratory response with resolution of the need for supplemental oxygen. However, owing to the low number of participants enrolled, the conclusions that can be drawn from this participant population are limited. Recruitment challenges are common in studies of patients with influenza, primarily due to the short high-incidence periods and opportunistic enrollment [10]. Influenza activity during the 2016–2017 season was generally moderate in both the United States and Europe, where the study was conducted, suggesting that the mild nature of the influenza season may have contributed to the low participant numbers [1, 11]. Eligibility criteria were relatively stringent with regards to concurrent conditions and medical history as this was the first study in hospitalized patients receiving IV danirixin, but participants had to be severe enough to require hospitalization for their influenza. Following efforts to enroll participants for the entire season, only 10 participants met eligibility criteria and could be enrolled. Although reasons for not enrolling subjects were not formally captured prospectively, information received from some investigators indicated that a large majority of potential participants did not meet eligibility criteria based on existing hospital records; this led to the conclusion that the probability of enrolling 300 participants in 2 more seasons would be very low. As such, the decision was made to terminate the study.

Current treatment guidelines for influenza requiring hospitalization recommend neuraminidase inhibitors as standard-of-care therapy, primarily oseltamivir and zanamivir [12]. However, the majority of clinical data for these antivirals come from outpatient studies of patients with mild, uncomplicated influenza [10]. Although observational studies have indicated that neuraminidase inhibitors may reduce severe clinical outcomes in patients hospitalized for influenza [13, 14], these findings have not been supported by clinical trials [15, 16]; and, as yet, no treatment has been approved based on controlled studies in this population. Treatment of hospitalized patients presents multiple challenges, given the severity of disease and potential for complications, secondary infections, and requirement for intensive care [17]. Thus, there is a clear need for efficacious therapies in this high-risk group. Data from the phase 2 trials of danirixin, although affected by low sample sizes, are therefore worthy of consideration, and further studies of this novel treatment are warranted.

In the current study, although the primary outcome of TTCR was observed to be longer in the majority (n = 7) of participants in the danirixin + OSV arms than in the 2 participants in the placebo + OSV arm, 10 participants form too small a group from which to draw conclusions. With such a small population, differences at baseline likely impacted study outcomes. A higher percentage in the danirixin arms had received prior oseltamivir or steroids, or both, as well as supplemental oxygen. Based on baseline characteristics, participants in the placebo group may have been less ill than in the danirixin groups at study entry. Although cross-trial comparisons should be interpreted with caution, a larger-scale phase 3 trial with over 600 participants hospitalized with influenza reported a median TTCR with placebo + OSV of 5.63 days; this is similar to that seen in the danirixin + OSV arms of this study [16].

IL-8 levels increased in nasal SAM samples in danirixin + OSV-treated participants, which may be indicative of target engagement, although variability and limited placebo samples for comparison prevent clear conclusions of the data. Interpretation of nasal wash analysis was hampered by the limited sample number, as samples were obtained from only 3 participants. Serum samples were taken for follow-up evaluation, but the samples were not tested for cytokines or chemokines due to the early termination of the study. This is in line with other clinical and preclinical studies, suggesting IL-8 increase as an indicator of CXCR2 antagonism [18–20].

The trend of decreasing serum EL-NE in both danirixin + OSV-treated groups in the current study, indicating fewer neutrophil-elastase–driven lung elastin degradation events, but interpretation is again limited by the low participant numbers.

The safety and tolerability of IV danirixin in our inpatient study was in line with the profile presented in outpatient studies in which danirixin was administered orally [8, 21]. One participant developed neutropenia 3 days post-treatment, based on laboratory results, which was not reported as an AE by the investigator. The 2 SAEs in this inpatient study (aggravated cardiac failure and COPD exacerbation) were not considered by the investigators to be related to study treatment, and there were neither fatal AEs nor any AEs leading to study withdrawal or discontinuation.

## CONCLUSIONS

Although the potential for interpretation leading to general conclusions in this study was clearly restricted by the low number of participants, this small phase 2 study evaluating the efficacy and safety of IV danirixin in combination with oral OSV showed that all 10 influenza-positive participants achieved the primary endpoint of TTCR that enabled hospital discharge or the resolution of vital signs that were maintained for 24 hours. The study also showed danirixin to have a safety and tolerability profile congruous with that observed in other studies of this selective and reversible antagonist of CXCR2.

## Supplementary Data

Supplementary materials are available at *Open Forum Infectious Diseases* online. Consisting of data provided by the authors to benefit the reader, the posted materials are not copyedited and are the sole responsibility of the authors, so questions or comments should be addressed to the corresponding author.

Supplementary_Figure_1

Supplementary_Material
