# Supplementary material for: Efficacy and Safety of Danirixin (GSK1325756) Co-administered With Standard-of-Care Antiviral (Oseltamivir): A Phase 2b, Global, Randomized Study of Adults Hospitalized With Influenza
Source: Open Forum Infect Dis. 2019 Apr 3;6(4):ofz163. doi: 10.1093/ofid/ofz163 (PMC6483311; doi:10.1093/ofid/ofz163)
Supplement: Supplementary_Figure_1 [file ofz163_suppl_supplementary_figure_1.pdf]

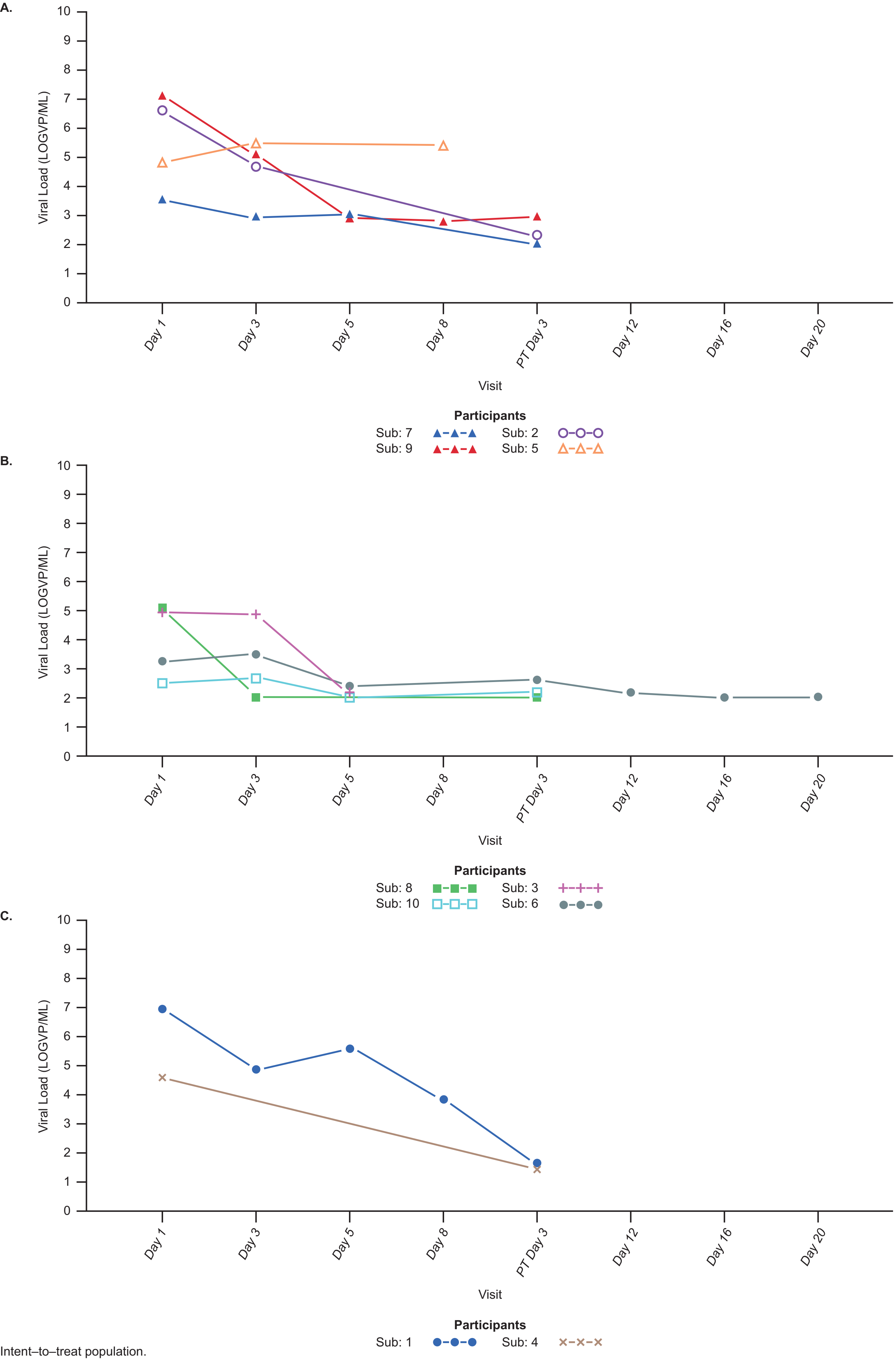

Intent–to–treat population.
Lower limit of detection was 2.05 Log^{10} vp/mL for influenza A and 2.83 Log^{10} vp/mL for influenza B
