# Supplementary material for: Efficacy and Safety of Danirixin (GSK1325756) Co-administered With Standard-of-Care Antiviral (Oseltamivir): A Phase 2b, Global, Randomized Study of Adults Hospitalized With Influenza
Source: Open Forum Infect Dis. 2019 Apr 3;6(4):ofz163. doi: 10.1093/ofid/ofz163 (PMC6483311; doi:10.1093/ofid/ofz163)
Supplement: Supplementary_Material [file ofz163_suppl_supplementary_material.docx]

**Supplementary Figure 1.** Viral Load in Participants Receiving (**A**) Danirixin 15 mg + OSV, (**B**) Danirixin 50 mg + OSV, and (**C**) Placebo + OSV

Intent-to-treat population.

Lower limit of detection was 2.05 Log_10_ vp/mL for influenza A and 2.83 Log_10_ vp/mL for influenza B.

**Supplementary Table 1.** Summary of influenza symptoms

|  | **Placebo + oseltamivir  75 mg (n=2)** | **Danirixin  15 mg + oseltamivir  75 mg (n=4)** | **Danirixin  50 mg + oseltamivir  75 mg (n=4)** |
| --- | --- | --- | --- |
| **Symptoms of influenza, n (%)** |  |  |  |
| Feverishness | 2 (100) | 4 (100%) | 4 (100%) |
| Cough | 2 (100) | 4 (100%) | 2 (50%) |
| Dyspnea | 2 (100) | 4 (100%) | 2 (50%) |
| Fatigue | 1 (50) | 4 (100%) | 3 (75%) |
| Nasal symptoms (rhinorrhea, congestion) | 2 (100) | 4 (100%) | 2 (50%) |
| Headache | 1 (50) | 4 (100%) | 1 (25%) |
| Myalgias | 0 | 4 (100%) | 2 (50%) |
| Nausea | 0 | 2 (50%) | 2 (50%) |
| Sore throat | 0 | 2 (50%) | 2 (50%) |
| Chills | 0 | 1(25%) | 1(25%) |
| Decreasing oxygen saturation | 0 | 0 | 1(25%) |
| Wheezing | 0 | 0 | 1(25%) |
| Vomiting | 0 | 1(25%) | 1(25%) |
| Diarrhea | 0 | 0 | 1(25%) |
| Prior exposure to OSV n (%) | 0 | 1 (25%) | 2 (50%) |
| Pre-Treatment Steroid use n (%) | 0 | 1 (25%) | 1 (25%) |
